# How acute myeloid leukemia (AML) escapes from FMS-related tyrosine kinase 3 (FLT3) inhibitors? Still an overrated complication?

**DOI:** 10.20517/cdr.2022.130

**Published:** 2023-04-28

**Authors:** Salvatore Perrone, Tiziana Ottone, Nadezda Zhdanovskaya, Matteo Molica

**Affiliations:** ^1^Hematology, Polo Universitario Pontino, S.M. Goretti Hospital, Latina 04100, Italy.; ^2^Department of Biomedicine and Prevention, the University of Rome “Tor Vergata”, Rome 00100 Italy.; ^3^Neuro-Oncohematology, Santa Lucia Foundation, I.R.C.C.S., Rome 00100, Italy.; ^4^Hematology, Department of Translational and Precision Medicine, Sapienza University, Rome 00161, Italy.; ^5^Hematology Unit, S. Eugenio Hospital, ASL Roma 2, Rome 00144, Italy.

**Keywords:** Acute myeloid Leukemia, FLT3, gilteritinib, midostaurin, TKI-inhibitor resistance, quizartinib, sorafenib, crenolanib

## Abstract

FMS-related tyrosine kinase 3 (FLT3) mutations, present in about 25%-30% of acute myeloid leukemia (AML) patients, constitute one of the most frequently detected mutations in these patients. The binding of FLT3L to FLT3 activates the phosphatidylinositol 3-kinase (PI3K) and RAS pathways, producing increased cell proliferation and the inhibition of apoptosis. Two types of FLT3 mutations exist: FLT3-ITD and FLT3-TKD (point mutations in D835 and I836 or deletion of codon I836). A class of drugs, tyrosine-kinase inhibitors (TKI), targeting mutated FLT3, is already available with 1^st^ and 2^nd^ generation molecules, but only midostaurin and gilteritinib are currently approved. However, the emergence of resistance or the selection of clones not responding to FLT3 inhibitors has become an important clinical dilemma, as the duration of clinical responses is generally limited to a few months. This review analyzes the insights into mechanisms of resistance to TKI and poses a particular view on the clinical relevance of this phenomenon. Has resistance been overlooked? Indeed, FLT3 inhibitors have significantly contributed to reducing the negative impact of FLT3 mutations on the prognosis of AML patients who are no longer considered at high risk by the European LeukemiaNet (ELN) 2022. Finally, several ongoing efforts to overcome resistance to FLT3-inhibitors will be presented: new generation FLT3 inhibitors in monotherapy or combined with standard chemotherapy, hypomethylating drugs, or IDH1/2 inhibitors, Bcl2 inhibitors; novel anti-human FLT3 monoclonal antibodies (e.g., FLT3/CD3 bispecific antibodies); FLT3-CAR T-cells; CDK4/6 kinase inhibitor (e.g., palbociclib).

## INTRODUCTION

In 1996, the first unexpectedly longer transcripts of FMS-like tyrosine kinase 3 (FLT3) were reported in the transmembrane domain through the juxtamembrane domain of the gene^[1]^. This represented a cornerstone discovery in acute myeloid leukemia (AML), since internal tandem duplications (ITD)-FLT3 mutations represent one of the most recurrent alterations, occurring in 22-32% and the remaining 8% including the tyrosine kinase domain (TKD), *de novo* AML^[2,3]^. Moreover, FLT3-ITD mutations, particularly those carrying a high FLT3-ITD mutant to wild-type allelic ratio when detected at initial diagnosis, seem to confer a short duration of remission and globally a worse prognosis^[4]^. The presence of FLT3-ITD appears to be strongly associated with hyperleukocytosis and high blast percentages in both peripheral blood and bone marrow at presentation^[5]^. From a morphologic standpoint, it has been suggested that AML with cup-like nuclei is associated with co-occurring mutations of both NPM1 and ITD- or TKD- FLT3^[6]^. Most importantly, we have an interesting class of drugs targeting FLT3, tyrosine-kinase inhibitors (TKI), which have an already long history in the treatment of this cancer. With the availability of 1^st^ and 2^nd^ generation inhibitors, the emergence of resistance or the selection of clones not responding to FLT3 inhibitors has become an important clinical dilemma, probably recalling the challenge of TKI in CML and ALL *BCR*: *ABL* positive. This review will try to summarize the evidence, from a clinical viewpoint, regarding the development of resistance to FLT3-inhibitors, their real clinical impact in AML patients, and strategies to obviate the development of resistance.

## FLT3 GENE AND ITS MOLECULAR FUNCTION

**Figure 1:**
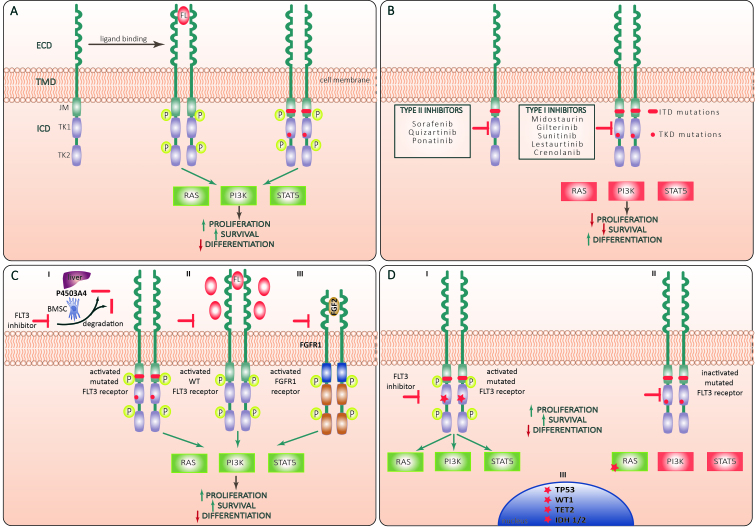
(A) Structure and activation of FLT3 receptor. FLT3 molecule is a tyrosine kinase receptor composed of an extracellular domain (ECD) that comprises 5 immunoglobuline-like repeats, a short transmembrane domain (TMD) and an intracellular domain (ICD) that includes a juxtramembrane (JM) domain followed by two tyrosine kinase (TK) domains (TK1 and TK2). Binding of the FLT3 ligand (FL) to ECD leads to formation of a ternary complex between FL and two receptor subunits with consequent conformational changes and trans-phosphorylation of TK and JM domains. The activated receptor exerts a tyrosine kinase activity against a series of adaptor and effector molecules leading to activation of Ras/Raf/MAPK (RAS) PI3K/Akt (PI3K) and Jak/STAT5 (STAT5) pathways promoting leukemic cell proliferation and survival and suppressing differentiation. Both FLT3-ITD and FLT3-TKD mutations lead to ligand-independent activation of FLT3 signaling; (B) types of FLT3 inhibitors. Type I FLT3 inhibitors interact with the ATP-binding site of the intracellular TK domain (TKD) when the receptor is in active conformation, whereas Type II inhibitors bind to the inactive form of the receptor, thus preventing its activation. Type I inhibitors inhibit FLT3 signaling in AML cells harboring ITD or TKD mutations, whereas Type II inhibitors are active against AML with ITD but not TKD mutations; (C) mechanisms of primary resistance to FLT3 inhibitors: Increased proliferation and survival and decreased differentiation of AML cells may be sustained through (I) Cytochrome P450 3A4-mediated degradation of FLT3-inhibitors by hepatocytes (liver symbol) and BM stromal cells (BMSC); (II) Restoration of FL/FLT3 signaling through compensatory overexpression of wild-type (WT) FLT3 receptor and FL; (III) Activation of common FLT3 and fibroblast growth factor receptor type 1 (FGFR1) downstream target pathways through the increased fibroblast growth factor 2 (FGF2)/FGFR1 signaling; (D) mechanisms of secondary resistance to FLT3 inhibitors: (I) Secondary resistance of FLT3 receptor to pharmacological inhibition may be conferred through newly acquired activating mutations of FLT3 TKD (red asterisk), and upregulation of pro-survival mechanisms through acquired activating mutations in RAS/MAPK pathway genes (II) and epigenetic modifiers like DNMT3A, TET2, IDH2 or transcriptional regulators like WT1 and TP53 (III). Green and red rectangles correspond to activated and inactivated FLT3 downstream signaling pathways, respectively. Yellow “P” circles indicate phosphorylated receptor domains. Green and red arrows indicate positive and negative regulation, respectively.

FLT3 (Fms-like tyrosine kinase 3) has strong similarities in its sequence with other members of the class III receptor tyrosine kinase (RTKIII) receptor family (FMS, platelet-derived growth factor receptor (PDGFR) α and β, and KIT)^[7]^. FLT3 is composed of an extracellular domain consisting of 5 immunoglobulin-like (Ig-like) domains, and by a cytoplasmic domain with a juxtamembrane domain and two intracellular tyrosine-kinase domains (TKDs)^[8]^. Physiologically, FLT3 is expressed over the cytoplasmic membrane of immature myeloid and lymphoid progenitor cells^[9]^ and is activated by its ligand FLT3L, which can be found in the cytoplasm of bone marrow stromal fibroblasts, T-cells, B-cells, and progenitors CD34+ [Figure 1]^[10]^. Noteworthy, FLT3 (CD135) is expressed on the surface of more than half of AML and seems associated with a worse outcome^[11]^. The binding of FLT3L to FLT3 activates the phosphatidylinositol 3-kinase (PI3K) and RAS pathways, producing increased proliferation and impaired apoptosis in cells. Moreover, activated PI3K stimulates downstream proteins like 3-phosphoinositide-dependent protein kinase 1 (PDK1), protein kinase B (PKB/AKT) and the mammalian target of rapamycin (mTOR), and PI3K activation hampers apoptosis through phosphorylation of the pro-apoptotic protein BAD, member of the BCL2-family. Moreover, the activation of RAS stimulates other downstream effectors like RAF, MAPK/ERK kinases (MEKs), extracellular-signal-regulated kinase (ERK), and the 90-kDa ribosomal protein S6 kinase (RSK). These downstream effectors can activate signal transducers and activators of transcription (STATs), which lead to the transcription of genes involved in cellular proliferation^[7]^. On the other hand, activating genetic mutations of FLT3 can produce the abnormal expression of a constitutively activated tyrosine kinase receptor in AML blasts, which is independent of the physiological FLT3L stimulus^[12]^.

Two kinds of FLT3 mutations have been individuated: FLT3-ITD and FLT3-TKD (including point mutations in D835 and I836 or deletion of codon I836)^[13]^. FLT3-ITD takes place between exons 14 and 15. When AML cells carry FLT3-ITD, PCR products generate a wild-type band and a larger ITD band after electrophoresis. D835 and I836 codons are encoded by the nucleotide GATATC, which forms the Eco-RV restriction site. The amplified products of wild-type FLT3 are digested into two bands by the Eco-RV enzyme, while amplified products with D835-mutations (FLT3-TKD) result in uncut bands, and this permits a traditional diagnosis^[14]^. Nowadays, several methods have been developed or adapted to help identify mutations of FLT3 and aberrant karyotypes (e.g., Multiplex-targeted next-generation sequencing)^[15]^, and moreover, next-generation sequencing (NGS) techniques for Measurable residual disease (MRD) detection are under development for clinical practice^[16]^. Especially in the pre-FLT3 inhibitors era, patients harboring FLT3-ITD had a worse prognosis in terms of OS and relapse-free survival^[17]^. Moreover, some studies suggested that a high Mutant-to-wild-type allelic ratio of FLT3-ITD was associated with a worse prognosis^[4,18]^. Other studies, however, found no increased risk of relapse in patients with FLT3-ITDhigh^[19]^. Other important determinants of prognosis in patients with FLT3-ITD are the karyotype status^[20]^ and NPM1mut which confers a more favorable outcome^[21,22]^. Indeed, patients harboring NPM1mut and FLT3-ITDhigh are classified as intermediate risk in ELN2017^[23]^. Different is the case for AML with FLT3-TKD; for these patients, a clear impact on prognosis is not established due to conflicting results^[24-30]^. However, in the new ELN2022 classification, probably due to the therapeutic benefit of FLT3 inhibitors in the new AML scenario, all patients with FLT3 mutation are classified as intermediate risk regardless of whether the mutation is detected alone or with other co-mutations. However, after the introduction of FLT3-inhibitors, this prognostic disadvantage of FLT3-mutations seems abrogated, improving levels of MRD^[31,32]^. MRD techniques to monitor FLT3 mutations are still being explored. In the next future, the improvement and the standardization of next-generation sequencing analysis could strongly increase and refine the opportunities for monitoring FLT3 mutations during treatment.

## CLASS OF DRUGS: FLT3-INHIBITORS

The class of drugs of FLT3-inhibitors can be categorized according to chronological order in 1^st^ generation (sorafenib and midostaurin) and 2^nd^ generation (gilteritinib, quizartinib, crenolanib). Another classification is according to the capacity of the TKI to link the active and inactive status of the mutated FLT3 (Type 1: midostaurin, gilteritinib) or the inactivated only status of FLT3 (Type 2: quizartinib, sorafenib)^[33]^. An important clinical implication is that Type 2 inhibitors at therapeutic concentrations are unable to inhibit FLT3-TKD, and, especially for the D835 mutation, this favors the active conformation of FLT3 and alters the binding of TKIs^[34]^.

Midostaurin is a first-generation TKI of class 1 and was the first drug to be approved for clinical use in FLT3-mutated AML in 2017^[35]^. Midostaurin has moderate activity as a single agent, as shown in 92 patients who achieved a reduction of bone marrow blasts in 71% of cases^[36]^. This evidence paved the way for a phase III trial of the association of 7 + 3 and midostaurin. From 2008 to 2011, the RATIFY trial enrolled a total of 717 patients harboring a mutated FLT3 (22.6% had the TKD mutation). The 4-year overall survival (OS) rate was 51.4% in the midostaurin group and 44.3% in the placebo group and the Median event-free survival (EFS) was 8.2 months in the midostaurin group *vs.* 3.0 months^[31]^. In a post-hoc analysis in patients with FLT3-TKD, the 5-year EFS rate was significantly extended in patients treated with midostaurin than in the placebo arm (45.2% *vs.* 30.1%; *P* = 0.044)^[37]^.

Gilteritinib, which belongs to the 2^nd^ generation of TKI, has more profound single-agent activity than 1^st^ generation drugs and has activity against both FLT3-ITD and TKD mutations^[38]^. Indeed, in phase I/II study with gilteritinib, 40% of patients achieved a response, with 19 (8%) reaching complete remission^[39]^. This fueled interest in a randomized phase 3 trial comparing gilteritinib against any chemotherapy (selected by investigators) in relapsed/refractory (R/R) AML with mutated FLT3. In the ADMIRAL trial, the median OS was significantly longer in patients treated with gilteritinib than among those receiving chemotherapy (9.3 *vs.* 5.6 months). The Overall response to gilteritinib was 67.6% and 25.8% for the chemotherapy arm^[17]^. These favorable results led to the approval of gilteritinib in the relapsed setting^[40]^. Interestingly, with a prolonged follow-up at 2 years after the first analysis, in the gilteritinib arm, the median duration of CR was 23 months and 16 patients remained on gilteritinib for a prolonged time^[41]^. Therefore, even if the majority of responding patients also underwent HSCT, there is a subgroup of patients that achieved a prolonged response to gilteritinib.

Sorafenib, a multitargeted TKI, is approved for hepatocellular carcinoma and renal cell carcinoma, but its off-label use in AML, especially after HSCT, is performed in many centers. Sorafenib is active only in FLT3-ITD AML with mild single-agent activity in the relapsed setting^[42,43]^. After HSCT, a synergism between sorafenib and allogeneic immunity after HSCT has been hypothesized^[44]^, even if the data are conflicting^[45]^. In a phase II study SORMAIN trial, 43 patients were randomized to receive a prophylactic treatment with sorafenib after HSCT and 40 patients to receive placebo. The 24-month RFS probability was 85% with sorafenib *vs.* 53.3% with placebo (*P* = 0.002)^[46]^. Moreover, different phase II studies explored the addition of sorafenib to standard induction chemotherapy, but failed to improve EFS and survival at the cost of increased toxicity^[47-49]^.

Quizartinib, a second-generation TKI, was specifically selected from a library of drugs to deeply inhibit FLT3-ITD, while it is inactive against FLT3-TKD^[50]^. It has clinical activity as a single agent in the relapsed setting, where 56% of FLT3-ITD-positive patients achieved a composite CR^[51]^. However, the FDA expressed concerns about the lack of improvement in EFS, so quizartinib was initially rejected. Recently, the FDA granted quizartinib Priority Review for newly diagnosed (ND) FLT3-ITD+ AML based on data from the QuANTUM-First study. QuANTUM-First was a pivotal trial randomizing quizartinib *vs.* placebo added to standard 7 + 3 backbone in FLT3-ITD+ AML patients. The OS was significantly prolonged in the quizartinib arm compared to the placebo arm. The median OS was of about 32 months with quizartinib *vs.* 15 months with placebo, while CR rates were 71.6% and CRi rates 64.9%^[52,53]^.

Crenolanib, compared to other drugs of the same class, demonstrates some attractive characteristics to target FLT3 mutations in AML. Being a potent type I pan-FLT3 inhibitor, crenolanib is also active in FLT3 TKD mutations^[54]^. As a single agent, in R/R AML Crenolanib showed 39% of CRi and 11% of PR among the 18 patients treated^[55]^, and in another study in 34 evaluable patients, 12% achieved CRi^[56]^. However, these responses to crenolanib were transient [Table 1].

**Table 1:** Clinical trials including targeted FLT3 Inhibitors in adult patients with AML

**Type of treatment**	**Therapeutic combinations including FLT3 inhibitors**	**Trial Phase**	**ClinicalTrials.gov identifier**	**AML^1^ setting**
**Chemotherapy + FLT3 inhibitors**	“7 + 3” + gilteritinib *vs.* “7 + 3”	3	NCT02236013	*de novo* AML^1^
	“7 + 3” + quizartinib *vs.* “7 + 3”	3	NCT02668653	*de novo* AML^1^ with mutated FLT3-ITD^2^
	“7 + 3” + crenolanib	2	NCT02283177	*de novo* AML with mutated FLT3
	CPX-351 + quizartinib	2	NCT04209725	R/R^3^ AML^1^ with mutated FLT3-ITD^2^
	“7 + 3” + crenolanib *vs.* “7 + 3” + midostaurin	3	NCT03258931	*de novo* AML^1^ with mutated FLT3
	“7 + 3” + crenolanib *vs.* “7 + 3”	3	NCT03250338	R/R^3^ AML^1^ with mutated FLT3
	HD-Ara-c^5^ + Mito^6^ + quizartinib	2	NCT03989713	R/R^3^ AML^1^ with mutated FLT3-ITD^2^
	CdA^7^ + ida^8^ + Ara-c^9^ + quizartinib	2	NCT04047641	*de novo* and R/R^3^ AML^1^
**HMAs + FLT3 inhibitors**	Aza^10^ or LD-Ara-c^11^ + quizartinib	1/2	NCT01892371	Phase I cohort: R/R^3^ AML^1^Phase II cohort: *de novo* and R/R^3^ AML^1^ with mutated FLT3
	Aza^10^ + gilteritinib *vs.* aza^10^	3	NCT02752035	*de novo* AML^1^ with mutated FLT3
**Maintenance with FLT3 inhibitors**	Crenolanib	2	NCT02400255	AML^1^ with mutated FLT3 post-transplant
	Gilteritinib *vs.* placebo	3	NCT02997202	AML^1^ with mutated FLT3 post-transplant
	Gilteritinib	2	NCT02927262	AML^1^ with mutated FLT3 post CR1^4^ achievement
**New molecules + FLT3 inhibitors**	Dec^12^ + ven^13^ + quizartinib	1/2	NCT03661307	*de novo* and R/R^3^ AML^1^ with mutated FLT3
	Ven^13^ + gilteritinib	1	NCT03625505	R/R^3^ AML
	Ven^13^ + quizartinib	1/2	NCT03735875	R/R^3^ AML with mutated FLT3
	Aza^10^ + ven^13^ + gilteritinib	1/2	NCT04140487	Phase I cohort: R/R^3^ AML with mutated FLT3Phase IIa cohort: *de novo* AML^1^
	Atezolizumab + gilteritinib	1/2	NCT03730012	R/R^3^ AML^1^ with mutated FLT3
	MTOR inhibitor (RAD001) + FLT3 inhibitor (PKC412)	1	NCT00819546	R/R^3^ AML^1^
	Milademetan (MDM2inh.) + quizartinib	1	NCT03552029	*de novo* and R/R^3^ AML with mutated FLT3
	“7+3” + GO^14^ + midostaurin	1	NCT03900949	*de novo* AML^1^ with mutated FLT3

AML: Acute myeloid leukemia; Ara-c: cytarabine; Aza: 5-azacitine; CdA: cladribine; CR1: first complete response; Dec: decitabine; GO: gemtuzumab ozogamicin; HD-Ara-c: high dose cytarabine; ITD: internal tandem duplication; Ida: idarubicine; LD-Ara-c: low dose cytarabine; Mito: mitoxantrone; R/R: relapsed/refractory; Ven: venetoclax.

## MOLECULAR MECHANISMS OF RESISTANCE

The heterogeneity of FLT3‐ITD mutations such as length, sequence, and duplication site is unique for every AML patient^[57,58]^, and this molecular diversity increases the complex biology of FLT3-ITD. As previously reported, FLT3-ITD length mutations are found in the JM domain of the gene in 70% of mutated AML cases, while in the remaining 30%, the duplication region can involve the TKD1 with a different prognostic impact^[30,59]^. However, the molecular mechanisms responsible for the differential signaling based on FLT3-ITD sites are not completely understood and can also influence the clinical outcome of patients [Table 2]. The use of FLT3 inhibitors in clinical practice showed a good response rate in mutated AML, both as frontline and relapsed/refractory therapy, but about 30%-40% of patients underwent relapse due to the acquisition or clonal evolution of gene alterations that drive therapy resistance^[17,60,61]^. Therefore, resistance to FLT3 inhibitors can be categorized as primary and secondary resistance. During the innate resistance, administration of FLT3 inhibitors at AML diagnosis may be impeded in its efficacy by FLT3 ligand, fibroblast growth factor 2 (FGF2) and stromal cytochrome P450 3A4 (CYP3A4) mediated manner. It is well known that FLT3-mutated AML cells also express high levels of the wild-type FLT3 receptor, and therefore, FL ligand expression is consequently increased during the administration of FLT3 inhibitors. FL ligand binds to the FLT3 receptor and, in turn, restores FLT3 and downstream MAPK signaling, allowing the FLT3-ITD mutated clone to survive during the induction and consolidation therapy^[62]^. In this scenario, FGF2 also plays an important role during TKIs resistance. Several studies have demonstrated that FGF2 is highly expressed in bone marrow stromal cells and its binding to FGFR1 receptor in FLT3-ITD mutated cells promotes resistance to FLT3 inhibitors through the activation of the downstream MAPK pathway^[63]^. Finally, loss of FLT3 inhibitors efficacy may arise due to insufficient drug concentrations in plasma, as a consequence of its rapid hepatic metabolism and in BM stromal cells, by cytochrome CYP3A4 enzymes^[64]^. Secondary resistance to FLT3-TKIs occurs through a variety of mechanisms, and in most cases, patients gain secondary resistance either by acquiring on-target or off-target abnormalities^[65]^. The majority of relapsed/refractory patients display on-target FLT3 alterations (26%), acquiring the D835, N676, F691, and Y862 mutations within the TK domain, the most common secondary events identified in AML patients treated with type II FLT3-TKIs^[66]^. Off-target mutations, which account for 16% of refractory patients, can occur *de novo* in primary FLT3-mutated clones or be gained by the evolution of other neoplastic clones. These cases are frequently characterized by gene mutations of epigenetic modifiers (DNMT3A, TET2, IDH2). Further genetic mutations leading to an off-target resistance after therapy with FLT3-TKI have been recently reported in genes of the RAS/MAPK pathway (13%), WT1 (7%), and TP53 (7%)^[66,67]^. There is hope that recognizing and understanding the mechanisms that produce FLT3-TKIs resistance will help provide better strategies for the rational design of new agents and, finally, lead to more effective treatment [Table 2].

**Table 2:** Proposed mechanisms of resistance to FLT3 Inhibitors in AML

**Mechanism**	**Description**	** References **
**Primary resistance**		
Cytochrome P450 3A4-mediated degradation of FLT3-inhibitors	Plasma levels of FLT3 inhibitors can be decreased through their enhanced degradation by hepatocytes and BMSC in Cytochrome P450 3A4-mediated manner	[64]
Restoration of FL/FLT3 signaling through compensatory overexpression of wild-type (WT) FLT3 receptor and FL	FLT3-mutated AML increases the expression of FL binding to WT FLT3 receptor and restores the downstream FLT3-MAPK signaling in WT and FLT3-ITD co-mutated cells	PMID: 27331411PMID: 21263155
Activation of common FLT3 and fibroblast growth factor receptor type 1 (FGFR1) downstream target pathways through the increased fibroblast growth factor 2 (FGF2)/FGFR1 signaling	FGF2 ligand highly expressed in BMSC binds to FGFR1 receptor in FLT3-ITD mutated cells and activates the downstream MAPK signaling shared by both receptor tyrosine kinases	[63]
**Secondary resistance**		
Newly acquired activating mutations of FLT3 TKD	Acquired mutations (D835, N676, F691 and Y862, A627 and others) within FLT3 TK domain confer resistance to different TKI and sustain activation of the downstream effectors	[66,71]PMID: 22858906PMID: 33780043
Acquired activating mutations in RAS/MAPK pathway genes	Activating mutations in RAS/MAPK pathway genes upregulate pro-survival and pro-proliferative mechanisms in leukemic cells	[66,67,69,74]
Acquired mutations in epigenetic regulators and transcriptional regulators	Acquired mutations in epigenetic modifiers like DNMT3A, TET2, IDH2 or transcriptional regulators like WT1 and TP53 upregulate pro-survival mechanisms and favor leukemic cells survival	[60,67,76]
GM-CSF and IL-3 maintain cell survival without rescuing proliferation	Cytokine-mediated resistance through GM-CSF and IL-3 is dependent on JAK kinase, STAT5, and pro-viral integration site of Moloney murine leukemia virus (PIM) but not MAPK or mammalian target of rapamycin signaling	PMID: 30944098
Novel ATM/mTOR pathway regulating oxidative phosphorylation	Marrow-mediated activation of ATM, in turn, upregulates oxidative phosphorylation via mTOR signaling. mTOR is required for the bone marrow stroma-dependent maintenance of protein translation	PMID: 36259537

AML: Acute myeloid leukemia; BMSC: BM stromal cells; FLT3: FMS-like tyrosine kinase 3; TKD: tyrosine kinase domain.

### Acquired mutation on *FLT3* gene

Several studies have demonstrated that TKIs drugs may have an impact on the clonal evolution of FLT3-ITD mutated AML by downregulating certain clones^[68,69]^. These findings emphasize the importance of repeated mutation analysis for FLT3-ITD to discriminate between patients in whom TKI may induce long-lasting remission, and from those in whom relapse may originate from subclones, which may carry a FLT3-wild type at diagnosis. As previously discussed, secondary TKD mutations, mainly reported at residues D835/F691 in FLT3-ITD mutated patients treated with TKIs, confer therapy resistance and poor outcomes^[70]^. In particular, at AML onset, type II of FLT3-TKIs have an effect on FLT3-TKD mutations, but secondary alterations in the TK domain during the disease progression may confer treatment resistance interfering with the inhibitory activity on FLT3-ITD mutated clones. Acquired alterations at D835, F691 and Y842 residues have been reported in patients who developed resistance to sorafenib or quizartinib, both type II-TKIs^[71]^. Although the acquisition of mutations in the TK domain of FLT3 is rarely reported in patients treated with gilteritinib and crenolanib (a type I-TKIs), the gaining of F691L alteration, defined as gatekeeper mutation of FLT3 gene, was frequently observed. This mutation confers substantial resistance to both type I and type II-TKIs^[65]^. The latter mutations occur in residues that directly interact with the TKIs and become prevalent in AML clones almost exclusively after treatment with the inhibitors^[72]^. Although in FLT3-mutated AML, the addition of midostaurin to standard “7 + 3” treatment regimen has been widely used in clinical practice, only 60% of patients achieved a CR, and almost half of these cases developed a relapse. Schmalbrock and colleagues^[16]^ provided novel biological features into the clonal evolution and mechanisms of resistance of FLT3-ITD-mutated AML exposed to midostaurin. They demonstrated that during the disease progression, almost 46% became FLT3-ITD negative and acquired mutations in MAPK pathways, conferring an additional proliferative advantage. By contrast, in AML cases that relapse with FLT3-ITD persistence, they showed a clonal selection of driver mutations in 11% of cases. FLT3-ITD clones persist in the remaining patients during relapse disease, indicating a failure of midostaurin inhibition activity.

### Intracellular pathways alterations

The phosphorylation of FLT3 receptor activates several downstream intracellular signaling pathways, such as RAS/MAPK, PI3K/Akt/mTOR, and JAK/STAT5, which are mainly implicated in the survival, proliferation, and differentiation of hematopoietic cells^[73]^. During the TKI treatment, clonal selection of cells characterized by activating gene mutations involved in RAS/MAPK pathway is often detected during progression in AML patients who received frontline midostaurin combination therapy^[69]^ or gilteritinib as monotherapy for relapsed/refractory disease in 2^nd^ line^[67,74,75]^. These results suggest that RAS mutations may drive the clonal evolution in relapsed/refractory AML that occurs independently from TKI type administration. Other mechanisms of off-target resistance during FLT3-TKI therapy have been recently described. Alotaibi and colleagues^[67]^ analyzed the mutational status of patients who relapsed after different FLT3 inhibitors and demonstrated that the most common gene alterations involved alterations in IDHs, NRAS, WT1, and TP53 genes. In particular, they identified in responding patients a higher occurrence of IDH2 alterations at diagnosis as compared to non-responder cases. This data suggests an advantage of combining targeted therapies in AML patients who harbored concomitant FLT3 and IDH2 mutations^[76]^. Instead, mutations of NRAS and IDHs genes often occur in leukemia subclones; TET2 alterations are described in FLT3 mutated clones and mainly enriched in crenolanib poor-responders AML. In particular, Zhang *et al.* reported that the mutation type of TET2 gene correlates with a different prognosis in patients treated with crenolanib, suggesting that TET2 truncation mutations may contribute to TKI resistance as compared to missense mutations that did not show a correlation with an unfavorable response to crenolanib^[60]^.

## CLINICAL RELEVANCE OF TKI RESISTANCE

It has been demonstrated that FLT3 inhibitor therapy promotes drug-resistant clonal populations that harbor secondary, on-target FLT3-mutations and that are prone to resistance to numerous TKIs in patients with relapsed AML who possess *FLT3-ITD* mutations^[77]^. Prolonged therapy with FLT3-TKIs can exert clonal pressure for the selection of drug-resistant sub-clones with additional mutations that facilitate leukemic proliferation regardless of the *FLT3* kinase’s activation state, as well as for sub-clones that are completely lacking FLT3 mutations; this is particularly relevant because relapsed AML is demonstrably a polyclonal disease^[69]^.

Sorafenib and midostaurin, FLT3 TKIs of 1^st^-generation, have in proportion a low selectivity for *FLT3* mutations. Nevertheless, the rates of response are augmented with midostaurin and relapse rates show a significant reduction when either drug is administered in association with frontline chemotherapy, like 3 + 7 induction therapy in patients with newly diagnosed (ND-AML), FLT3-^mut^ AML^[31]^. *FLT3-ITDs* are patient-specific, and due to the unique positioning and variable extension of the duplicated genomic sequence, there is a wide range of variability. This translates into unique peptide motifs derived from duplicate aminoacidic sequences within the *FLT3* gene. Therefore, many studies have tried to answer the question if diversity of *FLT3-ITD* exerts any effect on the clinical outcome of AML patients^[30,78-81]^. In a study conducted on 151 elderly AML patients who had received standard chemotherapy, Stirewalt *et al.* showed that the length of the ITD (40 *vs.* > 40 base pairs) had an effect on the patients’ long-term survival^[78]^. According to research by Kayser and colleagues, patients with AML who have *FLT3-ITD* placed into the first tyrosine kinase domain (TKD1) of the *FLT3*-gene present a poorer outcome than those who have *FLT3-ITD* in the juxtamembrane domain (JMD)^[30]^. However, a retrospective analysis in 260 patients with AML *FLT3-ITD* positive, which divided *FLT3-ITD* into 3 groups based on its position, was unable to confirm this finding, and just showed a statistical trend towards a link between a more C-terminal location of *FLT3-ITD* and worse survival, but without impacting on EFS^[79]^. However, Fisher *et al.* reported data favorable to the assumption that *FLT3-ITDs* located closer to the C-terminus of the FLT3 gene correlate with an adverse prognosis^[80]^. They showed that the localization of the ITD affected the percentage of remission following AML first-line chemotherapy, independently of the allelic burden^[80]^. Rucker *et al.* assessed the prognostic and predictive role of *FLT3-ITD* insertion site (IS) considering 452 subjects treated in the RATIFY trial, identifying 265 IS in the tyrosine kinase domain-1 (TKD1) and 43 IS in the juxtamembrane domain (JMD) by NGS^[81]^. Four-year OS probabilities significantly differed between JMDsole, JMD/TKD1, and TKD1sole, respectively; specifically, multivariate Cox models for OS and cumulative occurrence of relapse after HSCT identified TKD1sole as a negative prognostic factor^[81]^.

Gilteritinib and quizartinib, members of the second-generation FLT3 TKIs, show higher selectivity for FLT3 mutations. Indeed, when they are used as single agents in patients with FLT3-mutated R/R AML, both molecules exhibit clinical activity and have shown survival advantages over salvage approaches^[17,61]^. In comparison with quizartinib, gilteritinib showed activity for *FLT3-ITD* and *FLT3-TKD* mutations^[82]^. Nevertheless, secondary resistance to gilteritinib can arise via off-target mechanisms, such as the appearance of *NRAS* or similar mutations that activate MAPK signaling downstream of FLT3, as well as on-target FLT3 mutations at a gatekeeper FLT3 residue (F691L)^[40,74]^. As already discussed, gilteritinib has been approved as single-agent therapy for patients with FLT3-mutated R/R AML after positive results of ADMIRAL trial^[61,79]^. Patients randomized to receive 120 mg of gilteritinib showed a significantly longer median OS than those who received salvage chemotherapies and higher rates of CR^[17]^. Furthermore, Perl *et al.*^[39]^ confronted post-hoc the clinical outcomes in patients with R/R FLT3-mutated AML enrolled in CHRYSALIS and ADMIRAL trials^[17,39]^, with a view on those who were previously exposed to midostaurin or sorafenib against naive patients. Similar to those pre-treated, high rates of overall response emerged among patients treated with a FLT3 TKI before gilteritinib (CHRYSALIS, 42%; ADMIRAL, 52%) and patients without previous FLT3 TKI therapy (CHRYSALIS, 43%; ADMIRAL, 55%). Furthermore, in ADMIRAL study, a higher rate of response and a trend toward longer median OS was observed in the arm with gilteritinib *vs.* salvage chemotherapies in patients who had previously received a FLT3 TKI^[83]^. Therefore, these results encouraged the use of gilteritinib in FLT3-mutated R/R AML also after prior exposure to sorafenib or midostaurin.

## POSSIBLE STRATEGIES TO OVERCOME RESISTANCE

New therapeutic combinations are now being tested in order to potentially reduce the development of resistances to FLT3 inhibitors or the possible occurrence of new point mutations within the FLT3 gene during the therapeutic approaches; this would allow an increased rate of response to treatment and a lower cumulative incidence of relapse in FLT3 mutated patients.

A phase 1 study (NCT02236013) assessed the safety, tolerability, and efficacy of gilteritinib plus 7 + 3 induction, followed by consolidation chemotherapy with high-dose ARA-C, and then a period of maintenance with gilteritinib in adults with ND-AML. The study included 80 patients; the rate of composite CR was 81.8% for all the four doses and 81.6% for patients who received 120 mg of gilteritinib (that represented the maximum tolerated dose). The median follow-up for OS was 35.8 months, with a mOS for patients with FLT3-mutated disease that was not reached. Serious treatment-related adverse events (AE) took place in 10 patients; the most common nonhematologic AE of grade ≥ 3 were increased liver enzymes, pneumonia, and sepsis/bacteremia^[84]^. Recently, the MD Anderson group presented preliminary data from a phase II trial that evaluated the combination of gilteritinib with intensive chemotherapy (CLIA = cladribine, cytarabine and idarubicin) in ND-AML with *FLT3* mutation. Twenty-four patients were enrolled; 13 patients (54%) achieved a CR and underwent an allogeneic SCT^[85]^. Furthermore, treatment results for those patients with ND-AML, *FLT3*-mutated considered ineligible for a course of intensive chemotherapy are strongly disappointing. In a pooled analysis of the phase IB and phase III VIALE-A study (HMA + venetoclax), the median OS in FLT3 mutated patients was only 12.5 months, lower than what the 15 months achieved in FLT3 unmutated patients with this regimen^[86]^. Moreover, a phase III trial randomized (with a 2:1 ratio) untreated adults with FLT3 mutated AML unfit for intensive induction chemotherapy to gilteritinib (120 mg/day orally) and azacitidine (AZA + GIL) or azacitidine (AZA) alone. In the interim analysis, 123 patients were randomized (AZA + GIL, *n* = 74; AZA, *n* = 49). The median OS was not significantly different between the two arms (9.82 *vs.* 8.87 months, respectively; *P* = 0.753)^[87]^. As the median OS was the primary endpoint of the trial, the study was closed. Moreover, combination therapies to overcome resistance with FLT3 inhibitors and alternative resistance factors like apoptosis (BCL2) have been developed using venetoclax^[88]^. Preclinical data suggest that gilteritinib, inhibited the expression of BCL2A1 through the inactivation of STAT5 and alleviated TKI resistance of FLT3-mutated cell lines. Combining gilteritinib and venetoclax that suppresses BCL2A1 could improve the prognosis of AML with FLT3-ITD/D835 mutations^[89]^.

Some patients harbor *IDH* and *FLT3* mutations at the time point of diagnosis or experience a second mutation while undergoing treatment. Particularly *FLT3* mutations are linked to low IDH inhibitors response rates, and treatment-emergent *FLT3* mutations also seem to impart therapeutic resistance to these drugs. As a result, there is increased interest in researching combined therapies that target multiple pathways; however, there is currently no information available regarding the clinical experience of combined IDH inhibitors and FLT3 inhibitors therapy. A retrospective analysis identified 12 patients who received concurrent IDH and FLT3 inhibitors therapy, 11 of whom had R/R AML. The composite remission rate was 33%, and the ORR (CR + CRi + MLFS) was 42%, while the treatment combination was well tolerated^[76]^. Therefore, a notable proportion of R/R AML patients benefitted from concurrent FLT3 and IDH inhibitors therapy and studies are ongoing to investigate this promising combination.

In FLT3-ITD AML preclinical models, FLT3-ITD inhibition in combination with venetoclax exhibits impressive anti-tumor effectiveness and offers a solid molecular basis for clinical trials. However, the use of selective BCL-2 family inhibitors revealed a new function for BCL-2, BCL-XL, and MCL-1 in FLT3-ITD positive cells' *in vivo* survival, underscoring the necessity of targeting all three proteins for the most effective anti-tumor effect^[88]^. An open-label, phase 1b trial (NCT03625505) evaluated the combination of venetoclax and gilteritinib in R/R AML. Among the 54 patients enrolled, 38 patients (74.5%) achieved a response, with a median OS and a median duration of response of 10.5 and 5.6 months, respectively. In a post hoc analysis of the 30 analyzable patients who had achieved a CR with at least one follow-up MRD assessment, 17 (56.7%) achieved molecular clearance defined as FLT3 allelic burden < 10^-2^^[90]^. The MD Anderson group hypothesized that triplet therapy combining FLT3 inhibitors, venetoclax, and hypomethylating agents (HMAs) would further improve outcomes of FLT3 mutated patients. Therefore, they added FLT3 inhibitors to a regimen of 10-day decitabine with venetoclax in newly diagnosed (ND) and R/R FLT3 mutated patients. In ND patients, the composite complete remission (CRc) rate was 92%, with MRD negativity by flow cytometry in 56% and by next-generation sequencing (NGS) in 91% of responders. In R/R AML, the CRc rate was 62%, with MRD negativity rate by flow cytometry in 63% and by NGS in 100% of responders^[91]^. Therefore, triplet therapy with FLT3 inhibitors, venetoclax, and decitabine seems safe and an excellent frontline option for older patients with ND FLT3 mutated AML, and effective for R/R AML. A transition to allogenic transplant and post-transplant maintenance with FLT3 inhibitors would offer further improvement in long-term outcomes.

Although midostaurin and gilteritinib have been given FDA approval to treat patients with FLT3-mutations, the effectiveness of these treatments is still constrained by the fact that half of patients die during the first five years from diagnosis. Our actual understandings of the limits of TKIs highlight the demand for alternative therapeutic approaches. Although FLT3 antigen density on AML blasts is substantially lower than - for example, CD20 on lymphoma cells - and is not thought to be sufficient for producing powerful antibody-mediated effector functions, the membrane receptor FLT3 has also been studied as a target for immunotherapy using monoclonal antibodies. AML blasts and normal hematopoietic stem cells (HSCs), to a lesser extent, are selectively bound by the mouse anti-human FLT3 monoclonal antibody (mAb) 4G8. In preclinical models, 4G8 conferred selective reactivity against AML blasts with high FLT3 antigen density after Fc tuning^[92]^. Recently, T-cells engineered to express a FLT3-specific chimeric antigen receptor (CAR) were produced and it was shown that they confer robust reactivity against both AML cell lines and primary AML blasts, that express either wild-type FLT3 or FLT3-ITD. Furthermore, it was observed that treatment with crenolanib produced an increased surface expression of FLT3, particularly on *FLT3-ITD +* AML cells. Therefore, it enhanced the recognition by FLT3-CAR T-cells in vitro and *in vivo*^[93]^. Sommer *et al.* reported the preclinical evaluation of another off-the-shelf CAR-T cell construct targeting *FLT3*^[94]^. This CAR construct with single-chain variable fragments (scFvs) was directed against multiple *FLT3* extracellular epitopes and was investigated for its capacity to direct T-cell selectivity and effector function to FLT3-mut AML cells. AML initial blasts are eliminated by allogeneic FLT3 CAR T cells made from a group of voluntary donors of T-cells; however, these cells are also effective against mouse and human hematopoietic stem and progenitor cells, raising concerns about myelotoxicity. Authors demonstrated that rituximab-mediated reduction of FLT3 CAR T cells following AML eradication permitted bone marrow regeneration without compromising leukemia remission by using a surrogate CAR with an affinity for murine FLT3^[94]^.

Moreover, bispecific antibody cross-linking of FLT3 and CD3 demonstrated potent anti-leukemia activity against FLT3-mut cells. An anti-FLT3-CD3 immunoglobulin G (IgG)-based bispecific antibody (7,370) with a high affinity for FLT3 and a long half-life was developed to target FLT3-expressing AML blasts regardless of *FLT3* mutational status. In vitro and *in vivo* testing revealed that 7,370 exhibits picomolar potency against AML cell lines. Additionally, 7,370 was able to stimulate T cells from AML patients, refocusing their cytotoxic activity at low effector-to-target ratios on autologous blasts^[95]^.

**Figure 2:**
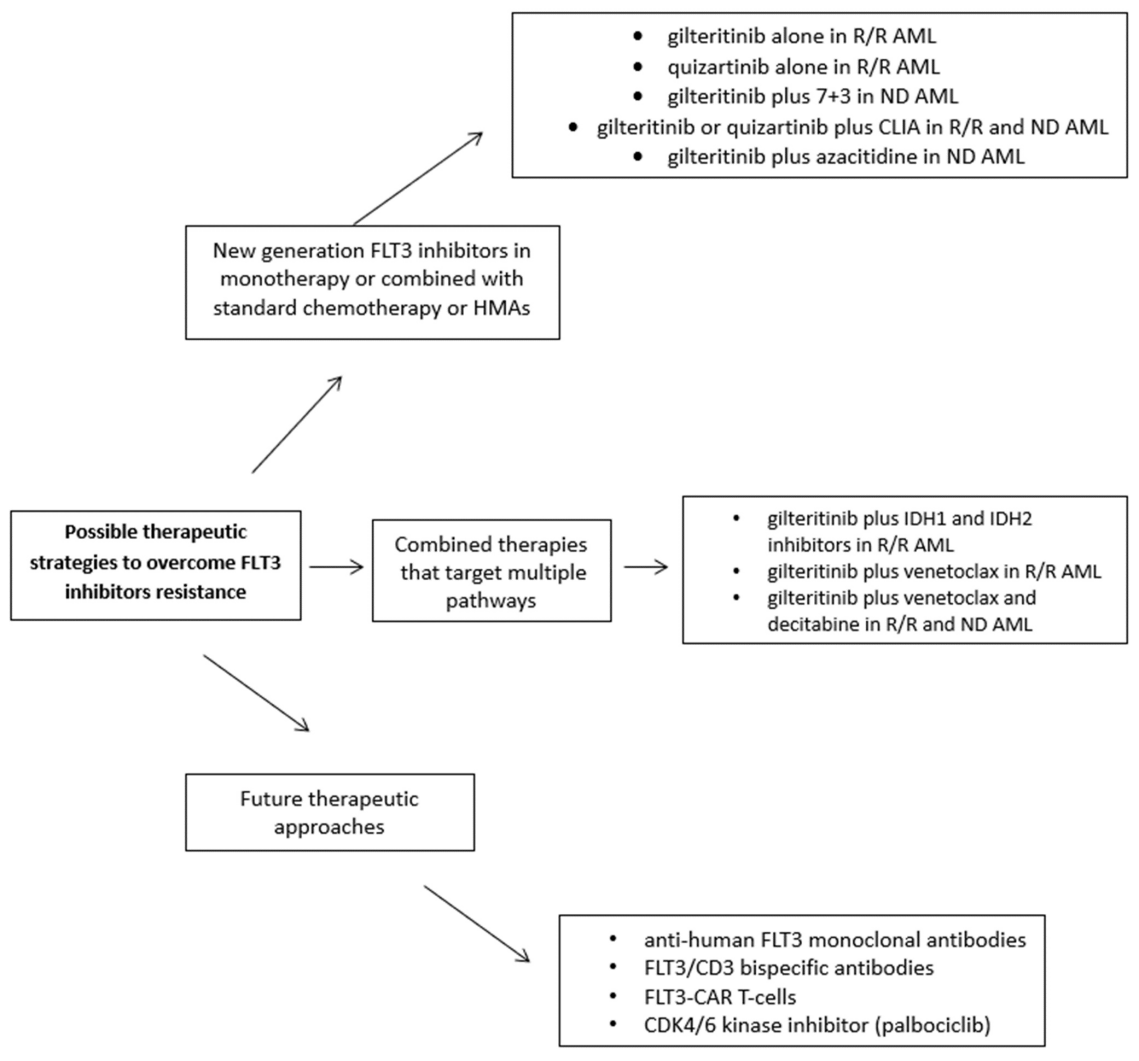
Possible strategies to overcome FLT3-inhibitor resistance.

Finally, cyclin-dependent kinase 6 (CDK6) is a protein that works as a transcriptional factor, regulating FLT3 and the serine-threonine kinase PIM1, a different step in the process of leukemogenesis^[96]^. Uras *et al.* found that FLT3-mutant AML cells are very responsive to palbociclib, one of several available inhibitors of CDK4/6 kinase^[97]^. They showed that the cell cycle kinase CDK6 is required for the viability of FLT3-dependent blastic cells and that FLT3-ITD could induce leukemogenesis [Figure 2]^[97]^.

## CONCLUSIONS

One of the most important modifications introduced with the current ELN2022 is that the *FLT3*-ITD allelic ratio is no more included in the ELN risk classification; as a consequence, AML with *FLT3*-ITD (without other adverse-risk genetic lesions) are now comprised into the group of the intermediate-risk and not the high-risk group, this independently from the allelic-ratio or the concurrent presence of *NPM1*-mut. The reasons for this modification are related to methodological difficulties in standardizing the assay adopted to measure the *FLT3*-ITD allelic ratio in different labs, and the improved effect of midostaurin-based chemotherapy on AML patients with *FLT3*-ITD without *NPM1*-mut^[55,98]^. Several mechanisms of resistance to available FLT3-inhibitors have been partly elucidated, but it is a process that acts underneath many biologic fields (e.g., the emergence of antibiotic-resistant strains in many bacteria), also with a clonal selection of resistant sub-clones of AML after protracted biological pressure from TKI-inhibitors. Nevertheless, in the ADMIRAL trial, patients who achieved a CR experienced a median duration of this response of 23.0 months, suggesting that the effect is protracted in a subset of patients. However, the cumulative effect of these drugs on patients with AML harboring FLT3-mutations has been demonstrated to be largely beneficial in clinical trials and current clinical practice. In Europe, we have only gilteritinib and midostaurin approved in clinical practice, so an exchange for a different TKI is not possible yet.

As a result, we reasoned, is resistance “Much Ado about nothing?” Probably, from the actual clinical perspective, mechanisms of resistance to FLT3-inhibitors are somehow overlooked. For patients obtaining a CR after therapy with gilteritinib, if they are transplant eligible, considering an early Allogeneic transplant seems appropriate because the duration of a CR is generally short-lived and gilteritinib may represent a bridge to transplant^[99]^. When gilteritinib is given after Allo-transplantation, it results in a significant improvement in overall survival. In brief, exposure to gilteritinib for a limited interval after allo-SCT enhances Graft-*vs.*-Leukemia effects against FLT3-ITD+ leukemia without exacerbating GvHD^[100]^.

Poor results have been reported in patients with R/R FLT3-ITD AML, who were treated with standard salvage chemotherapy and had a median OS of 5.5 months and 1- and 5-year OS rates of 25% and 7%, respectively^[101]^. On the contrary, the use of single-agent FLT3 inhibitors has improved survival over chemotherapy in the relapsed setting^[17]^. The path of FLT3 inhibitors is marked by possible strategies to combine different drugs to overcome the emergence of resistance^[91,93]^. We hope that these ongoing studies will benefit many patients in the future.

## DECLARATIONS

### Copyright

© The Author(s) 2023.
